# Prognostic impact of hypochromic erythrocytes in patients with pulmonary arterial hypertension

**DOI:** 10.1186/s12931-021-01884-9

**Published:** 2021-11-09

**Authors:** Panagiota Xanthouli, Vivienne Theobald, Nicola Benjamin, Alberto M. Marra, Anna D’Agostino, Benjamin Egenlauf, Memoona Shaukat, Cao Ding, Antonio Cittadini, Eduardo Bossone, Maria Kögler, Ekkehard Grünig, Martina U. Muckenthaler, Christina A. Eichstaedt

**Affiliations:** 1 Center for Pulmonary Hypertension, Thoraxklinik Heidelberg gGmbH at Heidelberg University Hospital, Röntgenstraße 1, 69126 Heidelberg, Germany; 2 Translational Lung Research Center Heidelberg (TLRC), German Center for Lung Research (DZL), Heidelberg, Germany; 3 Department of Translational Medical Sciences, “Federico II” University Hospital and School of Medicine, “Federico II” University, Naples, Italy; 4 IRCCS SDN, Naples, Italy; 5 Laboratory for Molecular Genetic Diagnostics, Institute of Human Genetics, Heidelberg University, Heidelberg, Germany; 6 Department of Cardiology, Division of Cardiac Rehabilitation‑Echo Lab Antonio Carderelli Hospital, Naples, Italy; 7 Department of Pediatric Oncology, Hematology, Immunology and Pulmonology, Molecular Medicine Partnership Unit (MMPU) Group Leader, University Hospital Heidelberg, Heidelberg, Germany

**Keywords:** Iron deficiency, Anemia, Hypochromic erythrocytes, Pulmonary arterial hypertension

## Abstract

**Background:**

Iron deficiency affects up to 50% of patients with pulmonary arterial hypertension (PAH) but iron markers such as ferritin and serum iron are confounded by several non-disease related factors like acute inflammation and diet. The aim of this study was to identify a new marker for iron deficiency and clinical outcome in PAH patients.

**Methods:**

In this single-center, retrospective study we assessed indicators of iron status and clinical parameters specifying the time to clinical worsening (TTCW) and survival in PAH patients at time of initial diagnosis and at 1-year follow-up using univariable and multivariable analysis.

**Results:**

In total, 150 patients were included with an invasively confirmed PAH and complete data on iron metabolism. The proportion of hypochromic erythrocytes > 2% at initial diagnosis was identified as an independent predictor for a shorter TTCW (p = 0.0001) and worse survival (p = 0.002) at initial diagnosis as well as worse survival (p = 0.016) at 1-year follow-up. Only a subset of these patients (64%) suffered from iron deficiency. Low ferritin or low serum iron neither correlated with TTCW nor survival. Severe hemoglobin deficiency at baseline was significantly associated with a shorter TTCW (p = 0.001).

**Conclusions:**

The presence of hypochromic erythrocytes > 2% was a strong and independent predictor of mortality and shorter TTCW in this cohort of PAH patients. Thus, it can serve as a valuable indicator of iron homeostasis and prognosis even in patients without iron deficiency or anemia. Further studies are needed to confirm the results and to investigate therapeutic implications.

**Supplementary Information:**

The online version contains supplementary material available at 10.1186/s12931-021-01884-9.

## Background

In the last decade, increasing attention has been paid to the role of iron metabolism in cardiovascular diseases [1, 2]. Iron deficiency can affect up to 50% of patients with pulmonary arterial hypertension (PAH) [3, 4]. Moreover, iron deficiency in PAH patients correlated with more severe disease, including reduced exercise capacity and worse survival [5–7]. While PAH guidelines suggest iron supplementation when needed [8, 9], iron supplementation trials in PAH patients provided inconclusive results in three smaller studies with around 22 PAH patients [6, 10, 11]. Only in two of these three studies the 6-minute walking distance increased significantly in previously iron deficient patients [10, 11]. Nevertheless, all studies presented improvements of study specific measurements such as quality of life [10], exercise endurance time [6] or right ventricular fractional area change [11]. In these studies, the definition of iron deficiency was based on the three parameters each, including serum ferritin, transferrin saturation (TSAT), and serum iron or hemoglobin concentration. Furthermore, treatment of iron deficiency should be managed with extreme caution since underlying conditions, such as infection and inflammation, that alter iron handling, put the patient at risk of having detrimental effects due to iron treatment.

Routinely, iron deficiency diagnosis relies on the TSAT and serum ferritin levels. However, these parameters can be altered by concomitant conditions such as inflammation and infections and, thus, not always accurately reflect the iron status [12]. In addition, the measurement of serum iron levels depends on the daytime and food intake [13]. Thus, these parameters are unreliable if not recorded under standardized conditions such as fasting. Moreover, TSAT is often not part of the routine laboratory assessment. Therefore, readily available, new iron deficiency indices which mirror the clinical status of the patient and which are less affected by inflammation are needed to facilitate therapeutic decisions.

Hypochromic erythrocytes may be such a new “biomarker” because they reflect upon the availability of iron in the previous 120 days and are both an early marker of anemia and an index of response to iron treatment [14]. Hypochromic erythrocytes mirror the current iron availability status in the bone marrow and erythropoietic system since they are produced as a consequence of iron deficient erythropoiesis [14]. Therefore, they are useful indicators of functional iron deficiency, providing values independent of concomitant inflammatory processes [15, 16].

The aim of the current study was to correlate routinely measured iron related parameters including the percentage of hypochromic erythrocytes with clinical data at baseline and during follow-up in particular with time to clinical worsening (TTCW) and survival in a large cohort of PAH patients.

## Methods

### Study design

This was a single-center, retrospective study analyzing TTCW and survival during yearly follow-up in PAH patients diagnosed and treated in the Centre for Pulmonary Hypertension between 04/2013 and 08/2017 at the Thoraxklinik Heidelberg gGmbH at Heidelberg University Hospital, Germany. This study evaluated data from incident PAH patients with at least one follow-up examination.

TTCW was defined as death, transplantation, hospitalization due to PAH, worsening of World Health Organization (WHO) functional class (FC) of at least one stage or 6-minute walking distance (6MWD) deterioration ≥ 15% compared to baseline. Clinical worsening was recorded either with date and type of worsening or death with date, cause and circumstances. The ethics committee of the Medical Faculty of Heidelberg University Hospital had no objection against the conduct of the study (internal number S-317/2020). The study complied with the Declaration of Helsinki in its current version.

### Study population

Inclusion criteria were incident patients aged ≥ 18 years with PAH (defined according to the European Society of Cardiology/European Respiratory Society pulmonary hypertension guidelines) [9] diagnosed by right heart catheterization, defined as a mean pulmonary arterial pressure ≥ 25 mmHg, pulmonary arterial wedge pressure ≤ 15 mmHg and pulmonary vascular resistance > 3 Wood Units. Patients with PAH and comorbidities were defined according to the published criteria from the 6th World Symposium on PH and the Cologne consensus conference [17, 18]. Patients with cardiac phenotype had PAH plus at least three of the following conditions: systemic arterial hypertension under medication, diabetes mellitus, treated for coronary artery disease in stable state disease, atrial fibrillation, enlargement of left atrium or obesity (defined by body mass index > 30 kg/m^2^). The included patients with pulmonary phenotype had normal or close to normal pulmonary function tests, no significant alterations in lung parenchyma in computed tomography of the lungs but low diffusion capacity for carbon monoxide < 45% of predicted or hypoxemia. Patients with pulmonary hypertension due to significant left heart disease or lung disease as defined by the guidelines were excluded [9] to avoid impact of other factors acting on iron metabolism such as anticoagulation or severe hypoxemia.

### Study investigations

The variables to investigate the clinical significance of iron deficiency were derived from routine laboratory assessments (ferritin, serum iron, hemoglobin, mean erythrocyte corpuscular volume, mean corpuscular hemoglobin, mean corpuscular hemoglobin concentration, red cell distribution width (RDW) and percentage of hypochromic erythrocytes, which contained less than 28 g/dl hemoglobin). Patients presenting with anemia or low ferritin values were treated with 1000 mg intravenous ferric carboxy-maltose if needed. Clinical parameters for characterization included demographic data, comorbidities, hospitalization data, vital signs, echocardiography, hemodynamics measured by right heart catheterization, spirometry, blood gas analysis, lung function, WHO FC, 6MWD, as well as the laboratory parameters for the assessment of renal function (creatinine, glomerular filtration rate).

### Statistical methods

Data including patient characteristics and the clinical parameters were presented using descriptive statistics with mean ± standard deviation and frequency tables. Frequency data, presented as n and %, were analyzed using the chi-square test. Survival was analyzed with Kaplan–Meier statistics and Cox regression analysis. For survival, date of initial diagnosis (i.e. right heart catheterization) was used as baseline and date of death due to any cause, date of lung transplantation or date of last patient contact when the patient was still alive were used for follow-up.

The prognostic value for survival and TTCW at initial diagnosis and survival one year after initial diagnosis were investigated by univariable and multivariable analysis with hemoglobin deficiency, RDW ≥ 15.7%, ferritin < 40 ng/ml, hypochromic erythrocytes > 2% and low iron as outcome variables. Hemoglobin deficiency was defined as < 12 g/dl in women and < 13 g/dl in men and low serum iron as < 12 µmol/l in women and < 13 µmol/l in men. Reference values are based on internal laboratory reference ranges or clinical routine cut-offs for iron supplementation. All variables identified with the univariable log rank tests as being significantly associated with survival (p < 0.05) were further analyzed using a multivariable Cox model.

Clinical characteristics between patient groups with better and worse survival for independent prognostic predictors were compared with Mann–Whitney U-Test. Non-parametric correlations were assessed with Spearmen’s correlation. Correlations of categorical variables were assessed with the Mann–Whitney test. P values  < 0.05 were considered statistically significant. All analyses were performed with IBM SPSS V 25.0 (IBM Corp. Released 2017. IBM SPSS Statistics for Windows, Version 25.0. Armonk, NY: IBM Corp.).

## Results

### Patient characteristics

Among 1522 right heart catheterizations performed at the Center for Pulmonary Hypertension, Thoraxklinik Heidelberg between April 2013 and August 2017, 150 patients had an invasively confirmed PAH diagnosis and a concomitant iron metabolism assessment. Table 1 shows baseline characteristics of the whole study cohort. Out of 150 PAH patients, 93 were female (62%) with a mean age of 63.3 ± 14.5 years. Forty-four patients were included with idiopathic PAH (29%), 4 with heritable PAH (3%), 50 patients with associated PAH (33%) and 52 patients were classified as PAH with comorbidities (35%, 33 cardiac and 19 pulmonary comorbidities). Right heart catheterization revealed severely increased pulmonary pressures with high pulmonary vascular resistances. Patients showed an enlarged right heart with mild impairment of right ventricular systolic function. On average patients displayed a normal renal function. Characteristics at one year follow-up are summarized in Additional file 1: Table S1. WHO FC and 6MWD remained largely unchanged within the first year after diagnosis.

**Table 1:** Baseline characteristics of the study cohort

	Whole cohort (n = 150) Mean ± SD or n and (%)	n
*Characteristics*		
Age, years	63.3 ± 14.5	150
Height, cm	166.3 ± 8.7	149
Weight, kg	78.0 ± 16.8	150
Body mass index	28.2 ± 5.7	149
Sex, female	93 (62)	149
6-min walking distance	331 ± 126	118
WHO functional class		130
II	25 (19.2)	
III	90 (69.2)	
IV	15 (11.6)	
*PAH classification*		
Idiopathic PAH	44 (29.3)	150
heritable PAH	4 (2.7)	
Associated PAH	50 (33.4)	
Other PAH*	52 (34.7)	
*PAH therapy*		
Monotherapy	98 (65.3%)	150
Double combination	50 (33.3%)	
Triple combination	2 (1.4%)	
*PAH medication*		
Calcium channel blocker	12 (8.0%)	150**
Endothelin receptor antagonist	62 (41.3%)	
Phosphodiesterase 5 inhibitor	116 (77.3%)	
Prostacyclin	2 (1.3%)	
Soluble guanylate cyclase stimulator	7 (4.8%)	
*Laboratory*		
Creatinine, mg/dl	1.05 ± 0.42	149
Glomerular filtration rate, %	69.2 ± 25.2	143
Iron, µg/dl	13.9 ± 6.7	74
Hemoglobin, g/dl	14.5 ± 1.8	149
Iron deficiency	40 (54.1)	74
Hemoglobin deficiency	16 (10.7)	149
Ferritin < 40, ng/ml	13 (16.0)	81
MCV < 83, fl	21 (14.1)	149
MCH < 27, pg/cell	17 (11.4)	149
MCHC < 30, g/dl	6 (4.0)	149
Hypochromic erythrocytes > 2%	21 (33.9)	62
*Diffusion capacity*		
DLCO SB, %	48.7 ± 22.3	130
*Echocardiography*		
Right atrial area, cm^2^	20.5 ± 6.3	142
Right ventricular area, cm^2^	24.4 ± 7.1	145
Systolic pulmonary arterial pressure, mmHg	63.6 ± 19.5	147
Tricuspid annular plane systolic excursion	1.9 ± 0.6	146
*Right heart catheter*		
Right atrial pressure, mmHg	8.0 ± 4.7	111
Mean pulmonary arterial pressure, mmHg	43.7 ± 12.4	150
Cardiac output, l/min	4.6 ± 1.3	134
Pulmonary arterial wedge pressure, mmHg	9.7 ± 3.1	143
Pulmonary vascular resistance, dynes*sec*cm^−1^	651 ± 323	142

*SD*  standard deviation, *PAH*  pulmonary arterial hypertension, *MCV*  mean corpuscular volume, *MCH*  mean corpuscular hemoglobin, *MCHC*  mean corpuscular hemoglobin concentration, *DLCO* diffusion capacity of carbon monoxide; *Other *PAH*  atypical PAH including 33 patients with cardiac and 19 with pulmonary comorbidities; **patients can belong to more than one category

### Anemia and iron metabolism

**Fig. 1:**
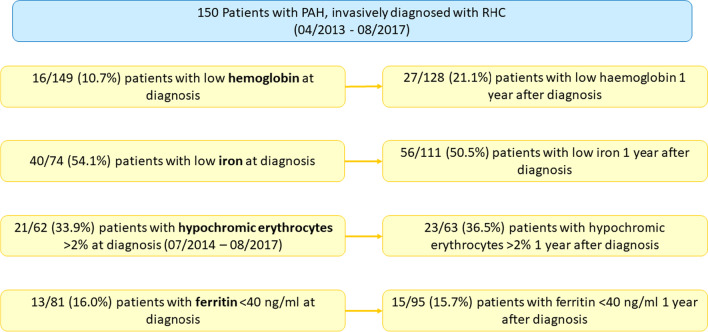
Iron metabolism and anemia in PAH patients at baseline and after 1 year. Within four years, 150 patients were included in the study. The percentage of patients with low hemoglobin doubled within a year, while the levels of low iron, hypochromic erythrocytes and ferritin < 40 ng/ml were comparable at baseline and 1 year follow-up

In the study cohort, 10.7% of patients presented with anemia, i.e., hemoglobin deficiency, at diagnosis (Table 1 and Fig. 1). The number of anemic patients almost doubled to 21.1% after 1-year of follow-up. Low serum iron was nearly unchanged between baseline (54.1%) and 1-year follow-up (50.5%). Likewise, hypochromic erythrocytes (33.9% at baseline vs. 36.5% after 1 year) and serum ferritin levels (16.0% at baseline and 15.7% after 1 year) showed similar values over time.

Since anemia and iron deficiency could also be caused by malignancies and gastrointestinal bleeding, we evaluated our patient cohort in this respect. Overall, a malignancy was reported in 12 patients: 9 have had a tumor before the diagnosis of PAH and were declared ‘tumor free’ at baseline; three developed a tumor after one, two and three years after diagnosis of PAH, respectively. The most frequent malignancies were breast cancer (6 patients), lung cancer (2 patients), other (4 patients). Only two of the patients had anemia at baseline, both had a past history of breast cancer and were tumor-free for ≥ 10 years before PAH diagnosis. Gastrointestinal bleeding occurred in 11 patients during follow-up (earliest after 6 months): in 7 cases due to gastrointestinal ulcers and in 4 cases due to angiodysplasia in the intestine. None of them had anemia at baseline.

### Survival and clinical worsening

**Fig. 2:**
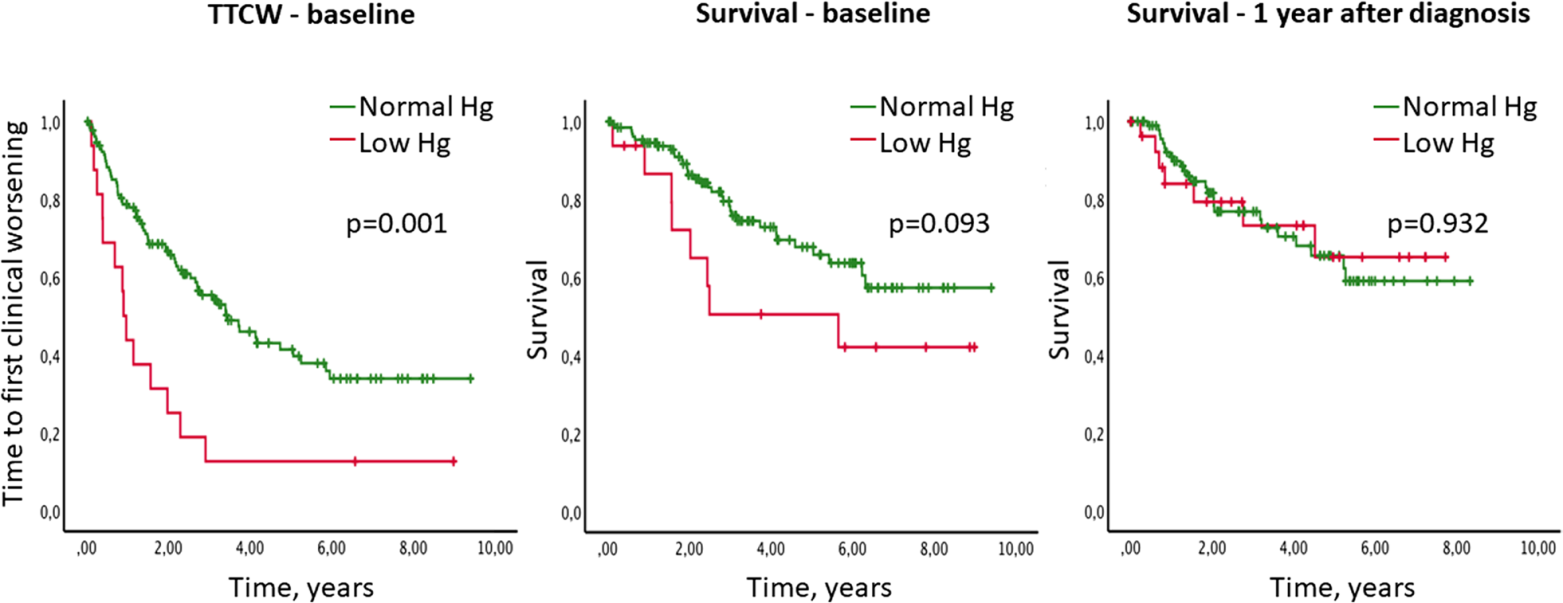
Survival and TTCW according to low hemoglobin levels. Low hemoglobin levels (red) were associated with shorter time to clincial worsening (TTCW, left) and showed a trend to predict survival at baseline (center) but not after 1-year follow-up in the univariable analysis (right). Green lines represent patients with normal hemoglobin (Hg) values, which corresponds to ≥ 12 g/dl in women and ≥ 13 g/dl in men

To identify parameters associated with TTCW and survival iron related parameters were assessed by Kaplan–Meier analyses (Table 2). Ferritin levels had no prognostic value for survival or TTCW. Hemoglobin deficiency was significantly associated with a shorter TTCW (p = 0.001) (Fig. 2) and showed a trend towards increased mortality in the group of patients with low hemoglobin at diagnosis (p = 0.093) in the univariable analysis. However, no association with survival and hemoglobin levels 1 year after diagnosis could be detected (Fig. 2).

**Table 2:** Uni- and multivariable analysis as predictors for survival and time to clinical worsening

	TTCW	Survival	Survival
	Baseline	Baseline	1 year
*Univariate analysis*			
Hemoglobin deficiency	0.001	0.093	0.932
Ferritin < 40, ng/ml	0.140	0.932	0.410
Hypochromic erythrocytes > 2%	< 0.0001	0.001	0.015
Iron low	0.900	0.722	0.030
*Multivariate analysis*			
Hemoglobin deficiency	–	–	–
Ferritin < 40, ng/ml	–	–	–
Hypochromic erythrocytes > 2%	0.0001	0.002	0.016
Iron low	–	–	–

**Fig. 3:**
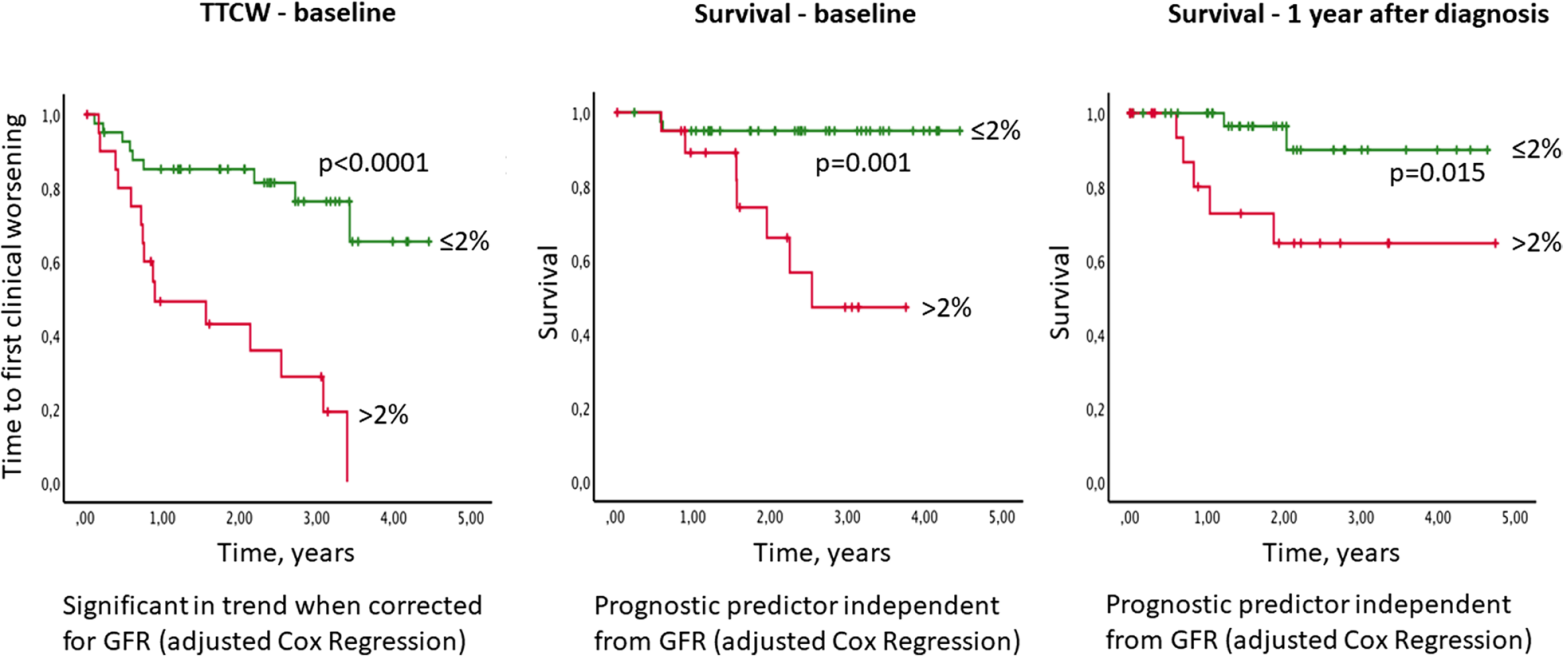
Survival and TTCW according to hypochromic erythrocytes rate. Patients with > 2% of hypochromic erythrocytes showed a shorter time to clinical worsening (TTCW, red lines, left) and had a worse prognosis at baseline (center) and after 1 year follow-up (right) in the univariable and mulitvariable analyses. The displayed p-values correspond to the univariable analysis. Green lines represent patients with ≤ 2% hypochromic erythrocytes

**Fig. 4:**
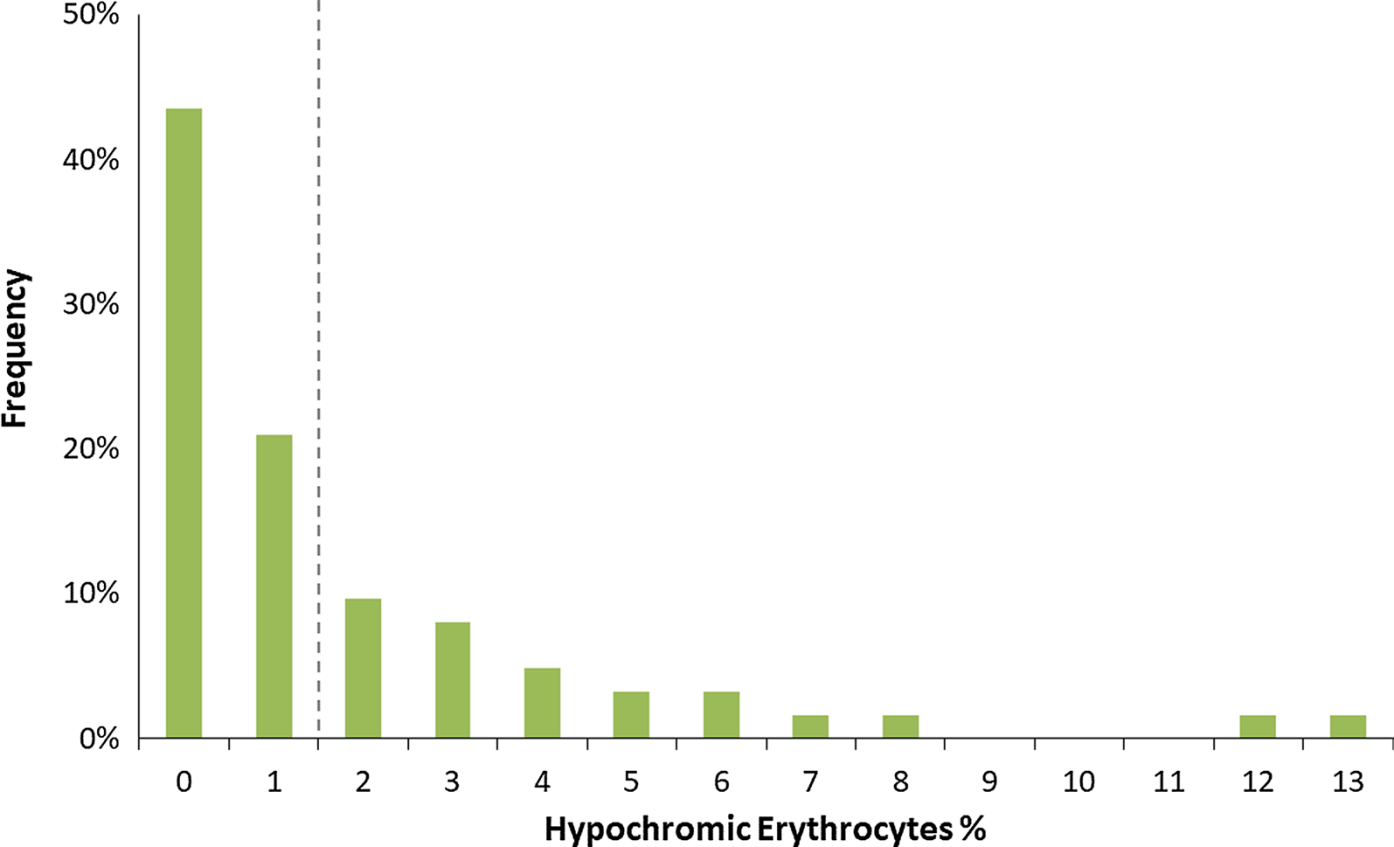
Distribution of hypochromic erythrocytes (%) in patients. The grey dashed line indicates the cut-off of 2% of hypochromic erythrocytes. The majority of patients had hypochromic erythrocytes < 2% (n = 41)

The presence of hypochromic erythrocytes > 2% at baseline resulted in a strong, independent prediction of TTCW (p < 0.0001) and survival (p = 0.001) in the univariable analysis (Fig. 3, Table 2). Likewise, also the presence of hypochromic erythrocytes > 2% detected 1 year after diagnosis was still associated with impaired survival (p = 0.015). Importantly, hypochromic erythrocytes were the only measurement which remained significant for TTCW and survival in the multivariable analysis (Table 3). The distribution of hypochromic erythrocyte levels in our patient cohort can be seen in Fig. 4. When the measure of RDW ≥ 15.7% [19] was added to the analysis hypochromic erythrocytes remained significant for TTCW (p = 0.011) together with low hemoglobin (p = 0.006), for survival at baseline low hemoglobin (p = 0.029) and RDW ≥ 15.7% (p = 0.011) were the strongest predictors, while for survival at one year hypochromic erythrocytes continued to be the only and highly significant predictor (p < 0.001).

**Table 3:** Characteristics of patients with normal and abnormal hypochromic erythrocytes at baseline

	Hypochromic erythrocytes at baseline		
	≤ 2%	> 2%	p-value*
	n = 41	n = 21	
	Mean ± SD or n (valid %)	Mean ± SD or n (valid %)	
*Characteristics*			
Age, years	62.4 ± 14.3	68.1 ± 15.2	0.106
Height, cm	166.9 ± 9.8	164.9 ± 9.1	0.227
Weight, kg	80.5 ± 16.7	80.9 ± 21.1	0.577
Sex, female	24 (58.5)	12 (57.1)	0.916
*PAH classification*			
Idiopathic PAH	19(46.3%)	3 (14.3%)	0.036
Associated PAH with connective tissue disease	6 (14.6%)	6 (28.6%)	
Portal hypertension	1 (2.4%)	0	
Associated PAH with congenital heart disease	0	2 (9.5%)	
Other PAH	15 (36.6%)	10 (47.6%)	
*Laboratory*			
Creatinine, mg/dl	1.1 ± 0.4	1.3 ± 0.5	0.054
Glomerular filtration rate, %	72.8 ± 28.1	57.4 ± 26.3	0.021
Iron, µg/dl	15.0 ± 5.5	13.2 ± 6.7	0.285
Hemoglobin, g/dl	15.0 ± 1.4	14.1 ± 1.8	0.048
Iron deficiency	10 (45.5%)	7 (63.6%)	0.455
Hemoglobin deficiency	0	4 (19%)	0.004
Ferritin < 40, ng/ml	3 (8.3)	3 (16.7)	0.380
MCV < 83, fl	1 (2.4%)	5 (23.8%)	0.007
MCH < 27, pg/cell	1 (2.4%)	3 (14.3%)	0.072
MCHC < 30, g/dl	0	1 (4.8%)	0.169
*Diffusion capacity*			
DLCO SB, %	55.7 ± 22.2	43.4 ± 21.8	0.022
*Echocardiography*			
Right atrial area, cm^2^	19.8 ± 6.5	22.7 ± 6.5	0.062
Right ventricular area, cm^2^	22.4 ± 5.4	24.9 ± 7.8	0.211
Systolic pulmonary arterial pressure, mmHg	61.0 ± 20.9	59.5 ± 19.7	0.855
Tricuspid annular plane systolic excursion	2.1 ± 0.6	2.0 ± 0.7	0.581
*Right heart catheter*			
Right atrial pressure, mmHg	7.1 ± 4.2	9.1 ± 3.9	0.053
Mean pulmonary arterial pressure, mmHg	40.2 ± 12.0	40.1 ± 9.2	0.743
Cardiac output, l/min	4.6 ± 1.2	4.6 ± 1.8	0.438
Pulmonary arterial wedge pressure, mmHg	9.8 ± 2.8	11.3 ± 3.1	0.045
Pulmonary vascular resistance, dynes*sec*cm^−1^	600.8 ± 363.2	590.6 ± 334.3	0.970

*PAH*  pulmonary arterial hypertension, *MCV* mean corpuscular volume, *MCH*  mean Corpuscular hemoglobin, *MCHC*  mean corpuscular hemoglobin concentration, *DLCO*  diffusion capacity of carbon monoxide

^*^Wilcoxon Mann–Whitney Test or chi-square test for frequency data

### Differences between patients with hypochromic erythrocytes ≤ 2% and > 2%

Patients with a higher rate of hypochromic erythrocytes (≤ 2% vs. > 2%), displayed a greater impairment of renal function (glomerular filtration rate 72.8 ± 28.1 vs. 57.4 ± 26.3 ml/min respectively, p = 0.021, Table 3), anemia (19% vs. none), and impairment of lung diffusion capacity (55.7 ± 22.2 vs. 43.4 ± 21.8%, p = 0.022). When renal insufficiency was defined as a glomerular filtration rate < 60 ml/min/1.73 m^2^ patients with hypochromic erythrocytes > 2% were not overrepresented (16/30 vs. 14/30; chi square test p = 0.06). Similarly, patients with oxygen pressure in capillary blood < 60 mmHg were not overrepresented in the group of patients with hypochromic erythrocytes > 2% (6/10 vs 4/10; chi square test p = n.s.).

No differences were found regarding right heart size or function in transthoracic echocardiography. Hemodynamics were comparable between the two groups except for a slightly higher pulmonary arterial wedge pressure in patients with more hypochromic erythrocytes (9.8 ± 2.2% vs. 11.3 ± 3.1%, p = 0.045). Absolute values of hypochromic erythrocytes showed a weak but significant correlation with right ventricular pump function (p = 0.02, Spearman correlation coefficient = 0.294). Interestingly, there was also a significant correlation between higher hypochromic erythrocytes and worse WHO FC, (Mann–Whitney Test: FC II vs. FC III p = 0.012, FC II vs. FC IV p = 0.001, FC III vs. FC IV p = 0.205). No correlations could be identified for hypochromic erythrocytes and age, gender, mean pulmonary arterial pressure, pulmonary vascular resistance, or right ventricular systolic pressure.

## Discussion

This is the first study identifying the biomarker “hypochromic erythrocytes higher than 2%” at baseline as a strong independent predictor for shorter TTCW (p = 0.0001) and worse survival (p = 0.002) in PAH patients. Increased numbers of hypochromic erythrocytes one year after initial diagnosis were also predictive of worse survival (p = 0.016). By contrast, ferritin and low serum iron were not able to predict mortality in PAH patients, although these parameters are commonly used in clinical routine to assess iron metabolism and to indicate iron supplementation therapy. Severe hemoglobin deficiency at baseline was significantly associated with a shorter TTCW (p = 0.001).

### Clinical impact of iron deficiency in PAH

Various combinations of iron markers, such as ferritin, TSAT or the soluble transferrin receptor are commonly used to define iron deficiency [20]. According to Sonnweber and colleagues, depending on the definition the prevalence of iron deficiency ranged from 11 to 75% in a cohort of 153 precapillary PH patients [20]. The lowest prevalence was based on the lowest cut-off values of TSAT together with serum ferritin while the highest prevalence resulted from less stringent, but routinely used cut-off values [20]. In the cohort analyzed here, 54% and 16% of PAH patients showed low iron and ferritin levels, respectively. Consistent with previous studies [5, 20] ferritin failed to predict TTCW or mortality in PAH. Overall, 10.7% of patients presented with anemia at diagnosis. The rate of anemic patients almost doubled to 21.1% after 1-year of follow-up, whereas hypochromic erythrocytes (33.9% at baseline vs 36.5% after 1 year) showed similar values over time. We speculate that ferritin, a marker for systemic iron availability, does not correlate with TTCW or mortality due to falsely high serum ferritin levels as result of inflammation and chronic kidney failure [21]. Impaired renal function often occurs in PAH and can increase ferritin levels due to a reduced renal excretion of the iron hormone hepcidin [21]. Hepcidin binds to the iron exporter ferroportin and triggers its degradation. This process significantly contributes to iron deficiency in serum as it diminishes dietary iron absorption and blocks iron release from macrophages that recycle aging red blood cells. As a consequence, ferritin levels rise despite diminished iron availability in the circulation [22]. This can falsely signal a normal iron status in PAH patients with impaired renal function. Furthermore, iron deficiency is involved in a circulus vitiosus in PAH. On one side, the upregulation of hepcidin in PAH leads to iron malabsorption, on the other side iron deficiency may counterfeit hypoxia, promoting pulmonary vasoconstriction leading to deterioration of the disease [23].

### Hypochromic erythrocytes as new “biomarker” reflecting iron status

Being less affected by inflammation status [24], hypochromic erythrocytes may provide crucial information for PAH patients since PAH is frequently associated with inflammatory conditions such as systemic sclerosis, HIV and porto-pulmonary hypertension [25]. The percentage of hypochromic erythrocytes has been previously suggested as a reliable marker of iron deficiency in kidney disease patients that is more reliable compared to TSAT and serum ferritin [15, 26]. Winkelmayer et al. showed that a level of hypochromic erythrocyte > 10% was associated with two-fold mortality over long term follow-up among kidney transplanted patients [27]. Increased percentage of hypochromic erythrocytes was strongly associated with iron deficiency, especially among patients with renal insufficiency [28]. Similarly, iron deficiency was associated with worse survival in PAH [12]. Rhodes et al. showed that RDW > 15.7%, an iron-dependent measure, is a prognostic parameter predicting survival in idiopathic PAH [19]. In our analysis with a mixed cohort of PAH patients RDW ≥ 15.7% was an independent predictor for survival at baseline, but not for TTCW or survival after one year. Until our study, the impact of the percentage of hypochromic erythrocytes on prognosis in PAH has not been investigated before. We showed for the first time that increased numbers of hypochromic erythrocytes reliably reflected iron deficiency even in PAH patients with normal ferritin. While only 64% of the patients with > 2% hypochromic erythrocytes showed low ferritin levels and only 19% were diagnosed with anemia, a worse survival could be identified in this cohort. The variability of the definition of iron deficiency in different conditions can lead to controversial indication for iron supplementation [23], depending on functional iron deficiency of each individual patient. Thus, hypochromic erythrocytes could be a reliable parameter of iron homeostasis and outcome even in the absence of anemia. Another advantage of hypochromic erythrocytes as new “biomarker” is its availability in the routine clinical practice.

Hypochromic erythrocytes might therefore be useful as a future therapeutic target in the context of iron supplementation therapy indication, outcome and monitoring.

### Limitations

Our study is limited by its retrospective nature. One major limitation was the absence of TSAT and soluble transferrin receptor levels as they were not measured in our patients within the routine work-up and could therefore not be included in the analysis. Moreover, no assessment of patients’ diet and nutrition was performed. Several biomarkers, such as soluble transferrin receptor index, growth differentiation factor-15 as well as inflammatory biomarkers such as interleukin 6 have been previously reported to be associated with the course of PAH [19]. Although the comparison of the new and the previously reported biomarkers would be interesting, it was not possible due to missing data. Moreover, we included no data of a non-disease control group in our analysis which could have characterized the new parameter in greater detail. However, we could analyze a large, clinically well characterized cohort of PAH patients. Currently we are performing a prospective study aimed at better characterizing iron metabolism in PAH patients and its correlation with clinical parameters (NCT04086537).

## Conclusions

Hypochromic erythrocytes > 2% independently predicted a reduced TTCW and survival. This new biomarker may be useful to assess the occurrence of functional iron deficiency with greater reliability compared to other routinely used parameters of iron status, such as ferritin, serum iron or hemoglobin. A parameter, which mirrors iron deficiency largely independent of an inflammatory state and shows prognostic capacity for TTCW and survival even in the absence of anemia could become a useful indicator of iron status and a valuable tool for indicating the need of iron supplementation and monitoring supplementation effects. Further studies are needed to confirm the results and to investigate therapeutic implications.

## Supplementary Information

**Additional file 1: Table S1.** Characteristics at one year follow-up of the study cohort.

## Data Availability

The datasets used and/or analyses from the current study are available from the corresponding author on reasonable request.

## Abbreviations

DLCO: Diffusion capacity of carbon monoxide
FC: Functional class
HIV: Human immunodeficiency virus
PAH: Pulmonary arterial hypertension
MCH: Mean corpuscular hemoglobin
MCHC: Mean corpuscular hemoglobin concentration
MCV: Mean corpuscular volume
n.s.: Not significant
RDW: Red cell distribution width
TSAT: Transferrin saturation
TTCW: Time to clinical worsening
WHO: World Health Organization
6MWD: 6-minute walking distance
