# Supplementary material for: Prognostic impact of hypochromic erythrocytes in patients with pulmonary arterial hypertension
Source: Respir Res. 2021 Nov 9;22:288. doi: 10.1186/s12931-021-01884-9 (PMC8579551; doi:10.1186/s12931-021-01884-9)
Supplement: Supplementary file 1 — Additional file 1: Table S1. Characteristics at one year follow-up of the study cohort. [file 12931_2021_1884_MOESM1_ESM.docx]

**Table S1. Characteristics at one year follow-up of the study cohort**

|  |  | **Whole cohort (n=150)**  **mean ± SD or n and (%)** | | |  |
| --- | --- | --- | --- | --- | --- |
|  |  |  |  |  | **n** |
| **Characteristics** | 6-minute walking distance | 343 | ± | 159 | 113 |
|  | WHO functional class |  |  |  | 130 |
|  | II | 24 |  | (20.2) |  |
|  | III | 82 |  | (68.9) |  |
|  | IV | 13 |  | (10.9) |  |
| **Echocardiography** | Right ventricular area, cm^2^ | 20.5 | ± | 6.1 | 121 |
|  | Systolic pulmonary arterial pressure, mmHg | 54.4 | ± | 20.4 | 122 |
|  | Tricuspid annular plane systolic excursion | 2.2 | ± | 0.5 | 121 |
| **Right heart catheter** | Right atrial pressure, mmHg | 7.6 | ± | 5.2 | 31 |
|  | Mean pulmonary arterial pressure, mmHg | 38.7 | ± | 15.0 | 31 |
|  | Cardiac output, l/min | 5.2 | ± | 1.0 | 31 |
|  | Pulmonary arterial wedge pressure, mmHg | 10.0 | ± | 4.4 | 31 |
|  | Pulmonary vascular resistance, dynes*sec*cm^-1^ | 462 | ± | 334 | 30 |
|  |  |  |  |  |  |

SD= standard deviation
